# PEX5 and Ubiquitin Dynamics on Mammalian Peroxisome Membranes

**DOI:** 10.1371/journal.pcbi.1003426

**Published:** 2014-01-16

**Authors:** Aidan I. Brown, Peter K. Kim, Andrew D. Rutenberg

**Affiliations:** 1 Department of Physics and Atmospheric Science, Dalhousie University, Halifax, Nova Scotia, Canada; 2 Department of Biochemistry, University of Toronto, Toronto, Ontario, Canada; 3 Cell Biology Program, Hospital for Sick Children, Toronto, Ontario, Canada; University of Notre Dame, United States of America

## Abstract

Peroxisomes are membrane-bound organelles within eukaryotic cells that post-translationally import folded proteins into their matrix. Matrix protein import requires a shuttle receptor protein, usually PEX5, that cycles through docking with the peroxisomal membrane, ubiquitination, and export back into the cytosol followed by deubiquitination. Matrix proteins associate with PEX5 in the cytosol and are translocated into the peroxisome lumen during the PEX5 cycle. This cargo translocation step is not well understood, and its energetics remain controversial. We use stochastic computational models to explore different ways the AAA ATPase driven removal of PEX5 may couple with cargo translocation in peroxisomal importers of mammalian cells. The first model considered is uncoupled, in which translocation is spontaneous, and does not immediately depend on PEX5 removal. The second is directly coupled, in which cargo translocation only occurs when its PEX5 is removed from the peroxisomal membrane. The third, novel, model is cooperatively coupled and requires two PEX5 on a given importomer for cargo translocation — one PEX5 with associated cargo and one with ubiquitin. We measure both the PEX5 and the ubiquitin levels on the peroxisomes as we vary the matrix protein cargo addition rate into the cytosol. We find that both uncoupled and directly coupled translocation behave identically with respect to PEX5 and ubiquitin, and the peroxisomal ubiquitin signal increases as the matrix protein traffic increases. In contrast, cooperatively coupled translocation behaves dramatically differently, with a ubiquitin signal that decreases with increasing matrix protein traffic. Recent work has shown that ubiquitin on mammalian peroxisome membranes can lead to selective degradation by autophagy, or ‘pexophagy.’ Therefore, the high ubiquitin level for low matrix cargo traffic with cooperatively coupled protein translocation could be used as a disuse signal to mediate pexophagy. This mechanism may be one way that cells could regulate peroxisome numbers.

## Author Summary

Peroxisomes are small organelles that must continually import matrix proteins to contribute to cholesterol and bile acid synthesis, among other important functions. Cargo matrix proteins are shuttled to the peroxisomal membrane, but the only source of energy that has been identified to translocate the cargo into the peroxisome is consumed during the removal of the shuttle protein. Ubiquitin is used to recycle peroxisomal shuttle proteins, but is more generally used in cells to signal degradation of damaged or unneeded cellular components. How shuttle removal and cargo translocation are coupled energetically has been difficult to determine directly, so we investigate how different models of coupling would affect the measurable levels of ubiquitin on mammalian peroxisomes. We find that for the simplest models of coupling, ubiquitin levels decrease as cargo levels decrease. Conversely, for a novel cooperative model of coupling we find that ubiquitin levels increase as cargo levels decrease. This effect could allow the cell to degrade peroxisomes when they are not used, or to avoid degrading peroxisomes as cargo levels increase. Regardless of which model is found to be right, we have shown that ubiquitination levels of peroxisomes should respond to the changing traffic of matrix proteins into peroxisomes.

## Introduction

Peroxisomes are single membrane organelles found in most eukaryotic cells [1]. They are involved in various anabolic and catabolic reactions including fatty acid oxidation, cholesterol biosynthesis, hydrogen peroxide metabolism, bile acid and plasmalogen synthesis [2]. Peroxisomal defects have been associated with serious genetic disorders such as Zellweger syndrome and neonatal adrenoleukodystrophy [3].

Peroxisomes are highly dynamic organelles, changing their numbers based on the specific metabolic needs of different tissues and cell types [4]. For example, in rodent livers, peroxisome numbers can rapidly increase two- to ten-fold in a matter of days by the activation of the receptor Peroxisome Proliferator-Activated Receptor-alpha (PPAR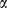) [5]. In yeast, changing the carbon source to oleic acid from glucose induces the rapid proliferation of peroxisomes [4].

Conversely, removal of peroxisome proliferators results in degradation of peroxisomes in mammalian cells with peroxisome numbers returning to basal levels within a week [6], [7]. Similarly, changing the carbon source from oleic acid back to glucose results in the decrease of peroxisome numbers in yeast within several hours [4], [8]. Peroxisomal degradation in mammals is mostly mediated by selective autophagy, the process of targeting cytosolic components to lysosomes for degradation (reviewed in [9], [10]) — called ‘pexophagy’ for peroxisomes. In pexophagy, superfluous or damaged peroxisomes are recognized by autophagic receptors that target peroxisomes either to autophagosomes or to lysosomes [11]. How peroxisomes are designated for degradation is not well understood. In mammalian peroxisomes, it has been hypothesized that sufficient ubiquitination of peroxisomal membrane proteins induces pexophagy by recruiting sufficient autophagy receptors such as NBR1 to peroxisomes [12], [13].

There are indications that any ubiquitinated membrane protein can recruit NBR1 [13], however the specific peroxisomal membrane protein(s) ubiquitinated to induce peroxisome degradation are not known. One candidate is the matrix shuttle protein PEX5, as preventing its recruitment to peroxisomes prevents NBR1 mediated pexophagy [12]. PEX5 is a cytosolic receptor that binds newly translated peroxisomal matrix proteins (cargo) through their peroxisome targeting sequence 1 (PTS1) [14]. PEX5, with cargo, is imported onto the peroxisomal membrane via its interaction with two peroxisomal membrane proteins PEX14 and PEX13 [15]–[17]. On the membrane PEX5 is thought to form a transient pore via an interaction with PEX14 to facilitate subsequent cargo translocation [18]. On the membrane, PEX5 is ubiquitinated by the RING complex, which is comprised of the peroxisomal ubiquitin ligases PEX2, PEX10, and PEX12. We call the RING complex, together with PEX13 and PEX14, an ‘importomer’. PEX5 can be polyubiquitinated, labelling it for degradation by the proteasome as part of a quality control system [19]–[21], or monoubiquitinated, labelling it for removal from the peroxisome membrane and subsequent recycling [22], [23]. Ubiquitinated PEX5 is removed from the membrane by the peroxisomal AAA ATPase complex (comprised of PEX1, PEX6 and PEX26) [24]. In mammals, monoubiquitinated PEX5 is deubiquitinated in the cytosol [25], completing the cycle and leaving PEX5 free to associate with more cargo.

The temporal coordination of cargo translocation, with respect to PEX5 ubiquitination by the RING complex and PEX5 removal by AAA, is not yet clear. This raises the basic question of how energy is provided to move cargo into the peroxisome. It has been suggested that there is no direct energy coupling, since it has been reported that cargo translocation happens before ubiquitination [26]. In this case, translocation of cargo would occur upon binding of PEX5 to the importomer. Subsequent removal of PEX5 would simply allow more PEX5-cargo to bind to the importomer, and the AAA ATPase is not necessarily involved in the energetics of cargo translocation. Conversely, an immediate or direct coupling of cargo import with PEX5 removal has been proposed in which energy for translocation would be provided by the AAA ATPase complex as it removes PEX5 from the membrane [27]–[29].

**Figure 1:**
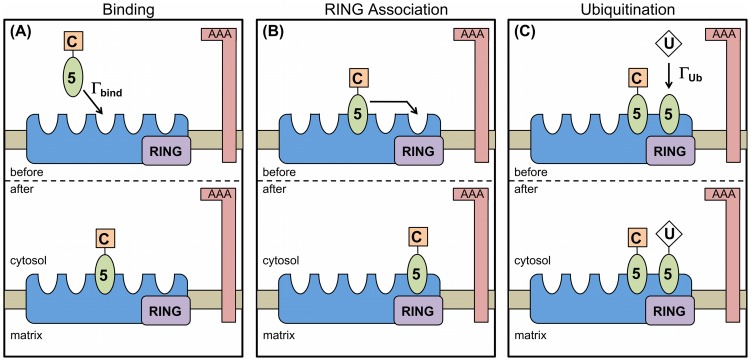
Illustration of model processes and associated rates that are shared between models. (A) PEX5 (green oval) associated with cargo (orange square) binds to available binding sites on a peroxisomal importomer (blue irregular shape) at a rate 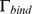. There are 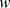 binding sites per importomer; here we illustrate 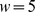. (B) If unoccupied, the RING complex site is immediately occupied by another PEX5 on the importomer. (C) The RING complex (purple rectangle) will ubiquitinate an associated PEX5 at rate 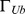. We generally allow only one ubiquitinated PEX5 per importomer. For (A), (B), and (C) the AAA complex is shown, and will participate in PEX5 export as described in Fig. 2.

**Figure 2:**
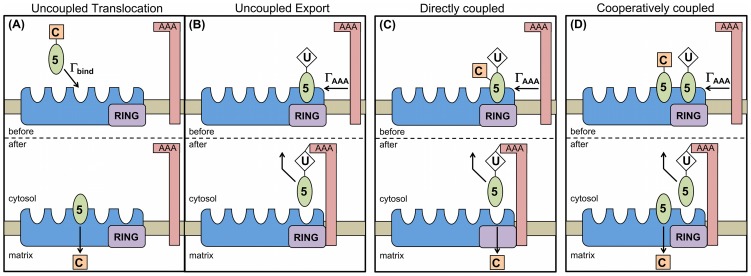
Illustration of translocation and export models and associated rates. (A) PEX5 (green oval) associated with cargo (orange square) binds to available binding sites on a peroxisomal importomer (blue irregular shape) at a rate 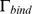. In uncoupled translocation, associated cargo is translocated spontaneously after binding to the importomer. (B) If translocation is uncoupled, then export of ubiquitinated PEX5 by the AAA complex at rate 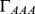 does not have a relationship with cargo translocation. (C) In directly coupled translocation, the cargo translocation occurs as the ubiquitinated PEX5 is removed from the importomer by the AAA complex at rate 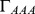. The PEX5 is shown simultaneously both cargo-loaded and ubiquitinated — this figure is meant to be illustrative; see Methods for discussion. (D) In cooperatively coupled translocation, the removal of PEX5 by the AAA complex (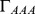) can only occur when coupled to the cargo translocation of a distinct PEX5-cargo in the same importomer. This always leaves at least one PEX5 associated with each importomer.

Using stochastic computational simulations, we have explored the implications of several models of how the PEX5 cycle couples cargo translocation with PEX5 removal by the AAA complex (see Figs. 1 and 2). The first, ‘uncoupled’, model corresponds to no direct or immediate coupling [26]. The second, ‘directly coupled’ model translocates PEX5 cargo as the same PEX5 is removed from the membrane by the AAA complex [27]–[29]. Our third, ‘cooperatively coupled’ model translocates PEX5 cargo when a different PEX5 is removed from the peroxisomal membrane. While this can be seen as a qualitative variation of directly coupled import, we show that this novel model behaves significantly differently than both uncoupled and directly coupled models of PEX5 cargo translocation.

We focus our modelling on accumulation of PEX5 and of ubiquitin on the peroxisomal membrane, as the traffic of PEX5 cargo in the cell is varied. This allows us to connect our models, of how PEX5 cargo translocation is coupled with PEX5 removal, with possible ubiquitin-regulated control of peroxisome numbers through pexophagy. Since both PEX5 levels and peroxisomal ubiquitination levels are accessible experimentally, this suggests an alternative approach to resolving how cargo translocation couples with PEX5 removal. Our modelling also shows that, regardless of what mechanism couples cargo translocation with PEX5 export, translocation coupling may have significant effects on ubiquitin levels of peroxisomes and so on regulation of pexophagy in mammalian cells. For example, both the uncoupled and directly coupled models lead to more ubiquitination with more cargo traffic. In contrast, the cooperatively coupled model leads to less ubiquitination with more cargo traffic. For cooperative coupling, this suggests a mechanism where lack of cargo results in the accumulation of ubiquitinated PEX5 on the peroxisomal membrane, thus leading to the degradation of underused peroxisomes.

**Figure 3:**
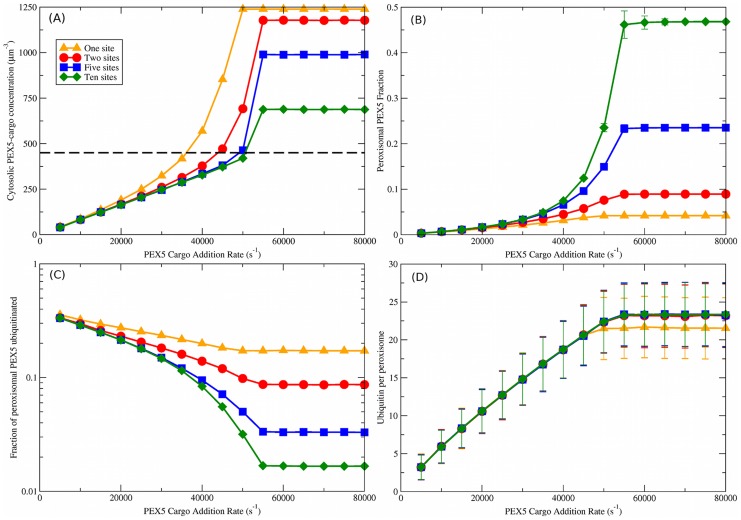
Uncoupled and directly coupled cargo translocation. Both uncoupled and directly coupled translocation models have identical PEX5 and ubiquitination behavior and so they are reported together. (A) cytosolic PEX5-cargo concentration vs. cargo addition rate, 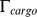. Different numbers of binding sites per importomer are shown from 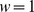 (orange triangles) to 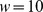 (green diamonds), as shown in the legend; the legend also applies to (B), (C), and (D). The dashed black line is the measured cytosolic PEX5 concentration of 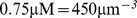 [43]. This is consistent with 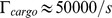 when 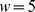. (B) Peroxisomal PEX5 fraction vs. 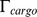. (C) Fraction of peroxisomal PEX5 that is ubiquitinated vs. PEX5 cargo addition rate, 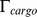. (D) Ubiquitin per peroxisome vs. 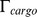. A characteristic increase of ubiquitination with 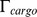 is seen that is largely independent of the number of binding sites 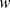. Vertical bars represent the standard deviation of observed values; error bars are smaller than point sizes.

**Figure 4:**
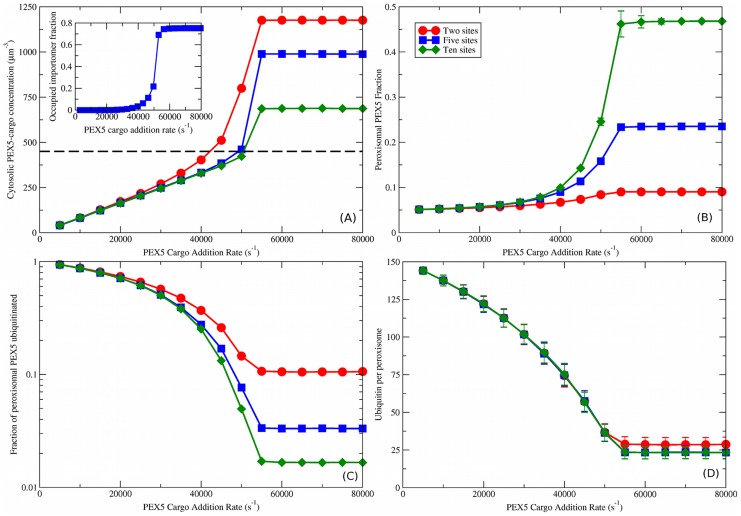
Cooperatively coupled cargo translocation. (A) Cytosolic PEX5-cargo concentration vs. PEX5 cargo addition rate, 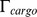. The dashed black line is the measured cytosolic PEX5 concentration of 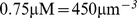 [43]. Inset shows the fraction of importomers that are fully occupied by PEX5 vs. PEX5 cargo addition rate, with five PEX5 sites per importomer and cooperative coupling. (B) peroxisomal PEX5 fraction vs. 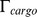 for cooperatively coupled cargo translocation. (C) Fraction of peroxisomal PEX5 that is ubiquitinated vs. 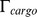. (D) ubiquitin per peroxisome vs. 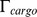. A characteristic decrease of ubiquitination with 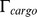 is seen that is largely independent of the number of binding sites 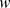. Different number of binding sites per importomer are shown from 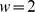 (red circles) to 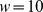 (green diamonds), as shown in the legend in (B). Cooperative coupling cannot function with 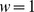, so that is not shown. Subsequent figures use 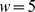 (blue squares). Note that the vertical scale of ubiquitin per peroxisome in (D) is much larger than in Fig. 3.

**Figure 5:**
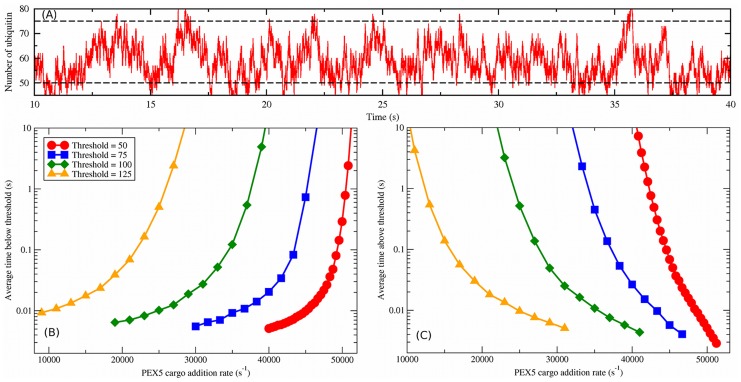
Ubiquitin thresholds for cooperative coupling. (A) Example time dependence of total peroxisomal ubiquitin for cargo addition rate 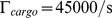, with the default number of peroxisomes (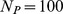) and importomers per peroxisome (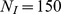). The characteristic timescale for fluctuations in the ubiquitination level is several seconds. Two possible threshold values are illustrated with dashed lines. (B) The average interval of time spent below a given threshold vs. 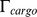 for thresholds as indicated by the legend, which also applies to (C). (C) The average interval of time spent above a given threshold vs. 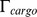.

**Figure 6:**
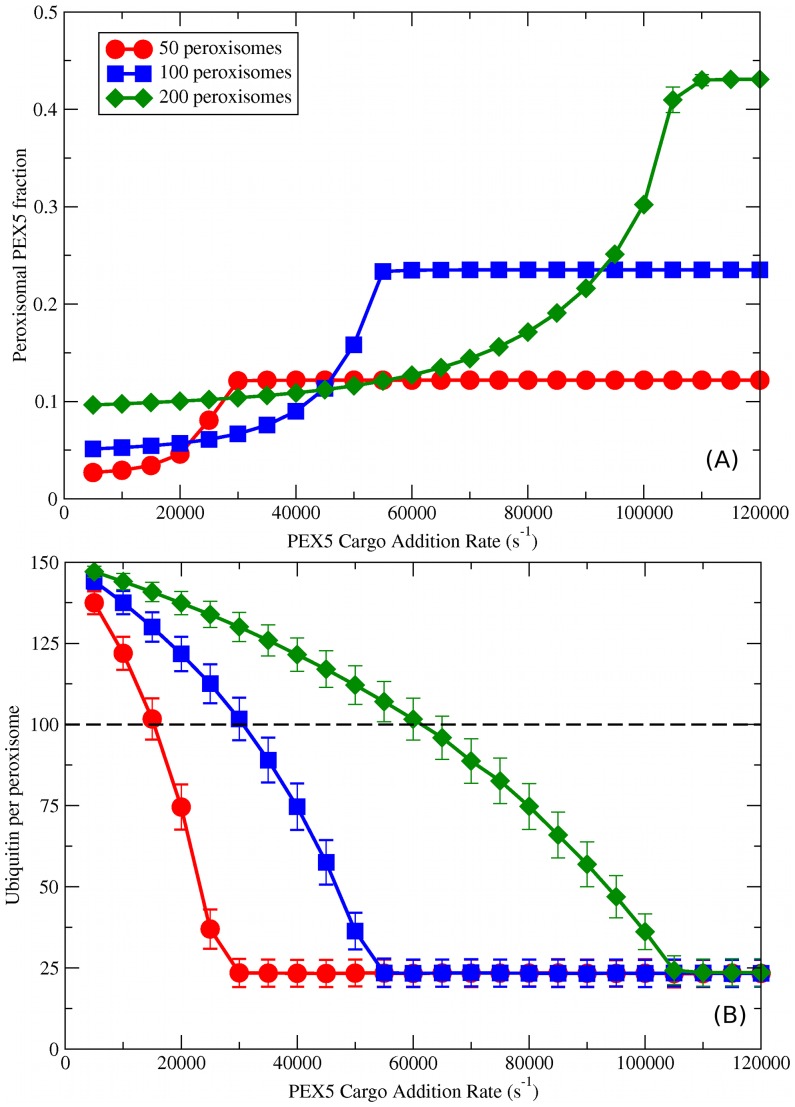
Peroxisome number variation for cooperative coupling. Here we investigate the effects of varying the number of peroxisomes (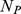, as indicated by legend in (A)) when the other parameters are kept constant (with 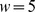 sites per importomer). (A) Peroxisomal PEX5 fraction vs. 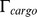 for cooperatively coupled cargo translocation. (B) Ubiquitin per peroxisome vs. 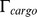. Horizontal black dashed line represents a possible ubiquitin threshold for peroxisome degradation.

**Figure 7:**
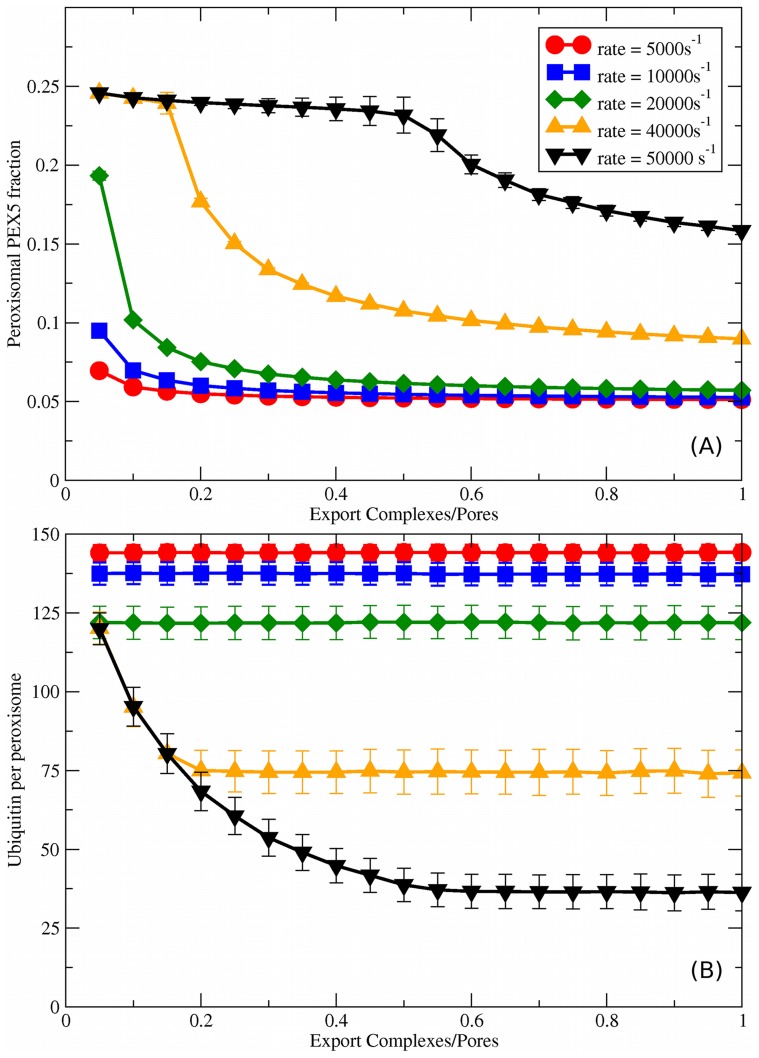
Export complex number variation for cooperative coupling. For cooperatively coupled systems with 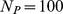, 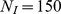, and 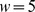 we vary the number of export complexes 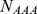, which directly scales the PEX5 export rate, 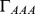. (A) Peroxisomal PEX5 fraction vs. stoichiometry of export complexes to importomers (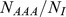). As shown in the legend, we consider different fixed rates of cargo addition, 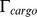; this legend also applies to (B). (B) Ubiquitin per peroxisome vs. 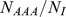, for the same set of 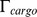.

Our figures are organized as follows. In the Methods section, Figs. 1 and 2 illustrate the three translocation coupling models. In the Results/Discussion section, Figs. 3 and 4 compares the behavior of these models. We then focus on cooperative coupling. We explore the fluctuations around possible ubiquitin thresholds for pexophagy with Fig. 5, and examine the role of numbers of peroxisomes with Fig. 6. Finally we investigate the effects of PEX5 export complexes with Fig. 7.

## Methods

### Translocation coupling models

We model four processes in the PEX5 cycle, each with an associated rate: the addition of peroxisomal matrix proteins, or cargo, to the cytosol (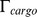), binding of PEX5-cargo to an empty site of an importomer (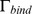), ubiquitination of a PEX5 at an importomer (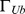), and export of ubiquitinated PEX5 from the importomer (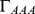). Binding of PEX5-cargo is illustrated in Fig. 1(A), association of PEX5 with the RING complex in Fig. 1(B), and ubiquitination of bound Pex5 in Fig. 1(C). RING association is assumed to be immediate relative to other modelled processes, and so has no associated rate. Fig. 2 illustrates the three distinct models of cargo protein translocation that we consider, discussed immediately below: uncoupled (Fig. 2(A) and (B)), directly coupled (Fig. 2(C)), and cooperatively coupled (Fig. 2(D)). These cargo translocation models differ in the details of how cargo translocation coordinates with AAA ATPase activity.

#### Uncoupled and directly coupled translocation models

Following reports that PEX5-cargo association with the peroxisomal membrane was ATP independent [30], [31], it was suggested that that cargo translocation may occur without concurrent ATPase activity [32]. We call this uncoupled translocation. AAA ATPase activity removes ubiquitinated PEX5 from the peroxisomal membrane [33]. Accordingly, the report that cargo translocation occurs before ubiquitination [26] supports an uncoupled model. We illustrate our uncoupled translocation model in Figs. 2(A) and (B), where cargo immediately translocates upon PEX5-cargo binding to an importomer.

Alternatively, it has been suggested that there may be a direct (immediate) coupling between the translocation of cargo bound to a membrane associated PEX5, and the AAA-driven removal of the same PEX5 from the peroxisomal membrane [28], [29]. Direct coupling is supported by results indicating that ATP is needed for cargo translocation [34] and that PTS2-targeted cargo translocation is directly linked to Pex18p shuttle removal in yeast [35]. We illustrate directly coupled translocation in Fig. 2(C), where cargo translocation occurs when ubiquitinated PEX5 is removed from the membrane by the AAA complex. For simplicity, the PEX5 in Fig. 2(C) is illustrated simultaneously both cargo-loaded and ubiquitinated.

In the uncoupled model individual PEX5-cargo translocate immediately upon membrane association, while in the directly coupled model translocation only occurs after both ubiquitination and AAA activity. Nevertheless, in both models each PEX5 binds, is ubiquitinated, and is exported by AAA activity at the same rates independently of the details of the cargo status. The dynamics of PEX5 and of ubiquitin are indistinguishable in these two models; only the precise timing of cargo translocation differs between them.

#### Cooperatively coupled model of cargo translocation and PEX5 export

We propose an additional possibility, in which more than one PEX5 is involved in the coupling between cargo translocation and AAA activity. This is our cooperatively coupled model of translocation, which we investigate for the simplest case of two PEX5. As illustrated in Fig. 2(D), this requires at least two PEX5 on an importomer — one of which has cargo, and the other of which is ubiquitinated. The import of the cargo of one PEX5 is coupled with the export of the second, ubiquitinated, PEX5. This is a variety of direct coupling between cargo translocation and AAA driven removal of PEX5 from the membrane [28], [29]. We further propose that the coupling of translocation and export is ‘tight’, i.e. export does not occur without coupled import. This would always leave at least one PEX5 per importomer, which is consistent with the *in vitro* observation of Oliveira *et al* [30] of a peroxisomal PEX5 population that remains even after prolonged incubation with ATP to promote AAA activity.

### Simulation details

We implement the models of the PEX5 cycle computationally using the Gillespie algorithm [36], for 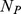 peroxisomes each of which has 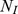 importomers, each with 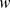 independent binding sites for PEX5-cargo, and all of which share a cytoplasmic pool of PEX5-cargo with concentration 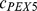. We track the number of bound PEX5 for every importomer, together with ubiquitination status of every bound PEX5. Association rates have not been determined experimentally, so we assume diffusion-limited association rates (see next subsection). This allows us to explicitly avoid fine-tuning of parameters. Parameter definitions and values for the quantitative model are summarized in Table 1.

**Table 1:** Model parameter definitions and values.

Variable	Description	Value/Eqn
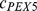	PEX5-cargo cytosolic concentration	variable
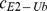	concentration of E2 enzyme with ubiquitin	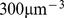
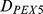	PEX5-cargo diffusivity	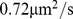
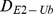	diffusivity of E2 enzyme with ubiquitin	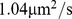
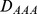	diffusivity of AAA export complex	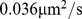
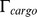	rate of addition of matrix proteins to cytosol	Varied
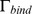	PEX5-cargo binding rate to empty importomer site	Eqn. 1
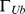	rate of ubiquitination of PEX5 at importomer	Eqn. 1
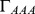	rate of export of ubiquitinated PEX5	Eqn. 2
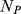	number of peroxisomes	100
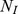	number of importomers per peroxisome	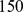
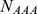	number of AAA export complexes per peroxisome	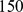
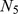	total number of cellular PEX5	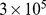
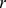	peroxisome radius	0.25 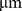
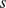	importomer radius	7.2 nm
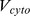	cytosolic volume	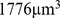

Shown are standard values used. Further discussion can be found in the Methods section.

In the model the total number of cellular PEX5 (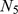) is held fixed, as is the cytoplasmic volume (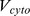), but the number of cytoplasmic PEX5 will vary as they cycle between the cytosol and the peroxisomes. We stochastically add cargo to the cytosol at fixed rate 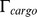. We assume the association rate is fast, and so we immediately bind cargo to any cytoplasmic PEX5 without cargo. Cargo accumulates in the cytosol if free PEX5 is not available. PEX5-cargo is removed from the cytosol when it binds to a peroxisome importomer [37] with a diffusion-limited rate 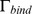 that depends on the number of importomers with available binding sites.

We generally assume that for each importomer there can be at most one ubiquitinated PEX5 by not allowing the RING complex to associate with more than one PEX5. We do not explicitly model RING complex motion or PEX5 motion within a given importomer, but once a ubiquitinated PEX5 has been removed from the peroxisome we allow ubiquitination of another PEX5 at a rate 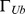. We have checked that our results are qualitatively unchanged, though with slightly higher ubiquitin levels, if we instead allow the RING complex to ubiquitinate all of the PEX5 associated with an importomer (see Fig. S1).

The AAA complex can remove ubiquitinated PEX5 from the peroxisomal membrane while the complex is transiently associated with the importomer [38]. This export occurs with a diffusion-limited rate 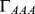 that depends on the number of export complexes, together with the number of importomers with ubiquitinated PEX5.

Every importomer is initially primed with a single PEX5 that is not ubiquitinated, since we do not have peroxisome or importomer biogenesis processes in our model. For most of our results, the system is run for ten simulated minutes, but data is not taken until after the first 10 simulated seconds; the simulation has reached steady state after this time and is run longer for improved statistics. The peroxisomal PEX5 fraction and ubiquitin per peroxisome are recorded every simulated 0.1s. Average times above and below thresholds in Figs. 5(B) and (C) were measured differently, as described below. Vertical bars indicate standard deviations. Statistical error bars are much smaller than the standard deviations, and are much smaller than the size of data points.

#### Diffusion-limited rates

Both cytosolic PEX5-cargo and E2-ubiquitin [39] diffuse to bind with peroxisomal importomers on the peroxisomal surface. The diffusion limited binding rate per importomer in terms of the appropriate cytosolic concentration 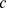 and diffusivity 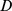, peroxisomal radius 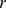, and number 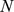 of available importomers each of radius 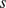 is [40]
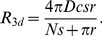
(1)We use this to determine PEX5-cargo binding rates, so that 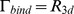 where 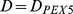 is the PEX5-cargo diffusivity, 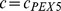 is the PEX5-cargo concentration, and 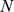 is the number of importomers with available binding sites — and both 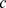 and 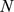 are time-dependent. We also use this to determine ubiquitination rates, so that 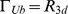 where 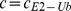, 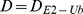, and 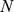 is the number of importomers without ubiquitinated PEX5 but with PEX5 — and only 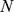 is time-dependent.

AAA ATPase complexes are thought to transiently interact with importomers [38], so we assume that they diffuse on the peroxisomal membrane. On a surface, each diffusing complex of diffusivity 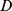 within a region of radius 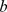 will be captured by an absorbing receptor of radius 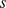 on average after a time [40]
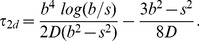
(2)We take the diffusion limited rate to be the inverse of this time, but proportional to the number 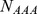 of AAA complexes, so that 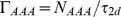 where 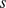 is the importomer radius and 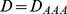. Assuming that the peroxisomal surface (sphere of radius 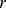) is evenly divided among 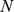 importomers that have ubiquitinated PEX5 then 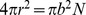 — i.e. 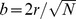. Unless otherwise noted, we assume that 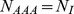, i.e. a 1∶1 stoichiometry of AAA complexes and importomers.

### Computational model parameterization

To approximate the diffusivity of PEX5 in the cytosol we note that the diffusion constant of EYFP in the cytosol has been measured at 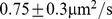 for NLFK cells and 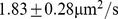 in HeLa cells [41]. We assume globular shape, and scale the diffusivity with the inverse radius, and the radius with the cube-root of the molecular mass. The molecular mass of PEX5 is 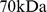 [42] with an additional 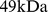 for cargo [43] giving 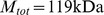. Using 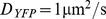 with mass 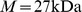, this gives 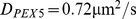.

Monoubiquitination of PEX5 in mammals is associated with the cytosolic UbcH5 family of proteins [39], which have a molecular mass of 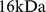 [44], [45]. Adding ubiquitin (8 kDa) we have 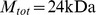, which scaled from YFP gives a diffusivity 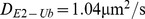. HeLa cell extracts have a UbcH5 concentration of 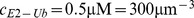 [46], assuming most of the E2 is activated with ubiquitin.

Diffusion in membranes of rat basophil leukemia (RBL) cells has a measured diffusion constant of 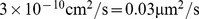 [47]. It has also been measured to be 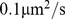 for mammals and 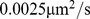 in yeast [48]. Most recently membrane diffusivity has been measured in yeast as 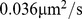 [49]. We use this most recent value, 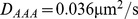, for the diffusivity of the export complex within the peroxisomal membrane.

The radius of a globular protein or protein complex can be approximated by 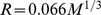 for R in nm and M in Daltons [50]. We estimate the size of an importomer complex by including both the docking machinery involving PEX14 and the RING complex, which have masses of 800 kDa and 500 kDa respectively [33]. For a total mass of 1300 kDa we obtain a radius of 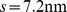.

Since very little is known about the population structure of peroxisomes, we use a fixed peroxisomal radius 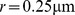 in the middle of the range of reported peroxisomal sizes (0.1–0.8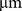 in diameter [51]). We use 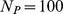 peroxisomes, unless otherwise stated, which for purposes of computational efficiency is slightly smaller than the average number of 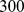 reported for mammalian cells [52]. For a spherical cell of radius 10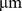, with 44.4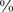 cytosol [43], then 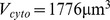. This is used to obtain concentrations of PEX5-cargo. A measured cytoplasmic concentration of PEX5, 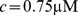 [43], corresponds to approximately 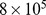 PEX5. We take a comparable but smaller number 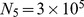, corresponding to the slightly smaller number of peroxisomes in our system.

We set the number of importomers per peroxisome 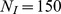. With 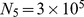, this works out to 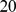 PEX5 per importomer when 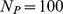. This is much more than the number of possible PEX5 binding sites 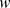 per importomer that we explore, which reflects the small proportion of PEX5 typically reported on peroxisomes [53].

#### Threshold calculations

For the numerical computation of average time intervals above and below specific ubiquitination thresholds, shown below in Figs. 5(B) and (C), we found that the averages are biased towards smaller intervals in short simulations. Accordingly, data was taken until averages no longer increased with increased sampling, where we increased the number of intervals averaged in factors of ten. For a threshold of 50 ubiquitin, this required 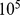 intervals and for all other thresholds this required 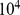 intervals.

We also found that the distribution of time-intervals either above or below specific ubiquitin thresholds was bimodally distributed. Fig. S2 shows an example distribution of recorded times spent below a threshold of 100 ubiquitin. We found that all distributions have a short-time peak below 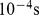 and another above 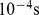. The shorter peak arises from many rapid crossings of the threshold (see Fig. 5(A) for an example trajectory) and are unlikely to be resolvable experimentally or be relevant to autophagy regulation. Accordingly, interval times below 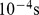 were not included in the computation of average intervals.

## Results/Discussion

### Uncoupled and directly coupled PEX5 and ubiquitin dynamics

We first examined uncoupled and directly coupled models of protein translocation coupling, shown schematically in Figs. 2(A)–(B) and (C), respectively. As mentioned above, the dynamics of PEX5 and ubiquitin are indistinguishable for these two models. We consider different number of sites 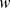 on each importomer for PEX5 binding in Fig. 3, guided by studies showing distinct [18], [54], [55] PEX5∶PEX14 stoichiometries on the peroxisomal surface —— as well as explicit suggestions of multiple PEX5 sites at the importomer [30]. For each 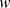, we vary the cargo addition rate 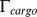 and consider both PEX5 populations and ubiquitination levels.

As shown in Fig. 3(A), the cytosolic PEX5-cargo concentration increases approximately linearly for small 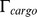 then sharply increases before reaching a constant plateau at larger 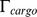. The linear regime arises from a dynamic balance between cytosolic concentration and concentration-dependent binding to peroxisomes through 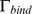. The plateau arises from saturation of the PEX5 cycling rates, together with complete binding of cytoplasmic PEX5 with cargo. The steep rise before the plateau occurs when the PEX5 cycling becomes rate limited by PEX5 removal through 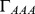, and coincides with sharply increased peroxisomal PEX5 fraction (see below) — essentially more and more importomers are fully occupied by PEX5 and so cannot contribute to PEX5-cargo binding (see Fig. 4(A) inset). Increasing the number of binding sites per importomer, 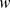, decreases the cytosolic fraction of PEX5-cargo. The experimentally measured value of 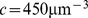 (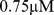 [43]) is consistent with all 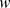, and roughly corresponds to where the PEX5-cargo concentration sharply increases due to saturation of importomer binding sites (around 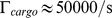).

Mirroring cytosolic PEX5-cargo concentrations, Fig. 3(B) shows that the peroxisomal PEX5 fraction also increases with 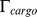. The mutual increase is possible with a fixed number of PEX5 (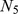) at the expense of the reservoir of cytosolic PEX5 that is not associated with cargo. PEX5 accumulates on the peroxisome because of the increasing binding rate due to increasing cytosolic PEX5-cargo concentrations. Increasing the number of binding sites per importomer 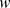 increases the peroxisomal fraction of PEX5. Fig. 3(C) shows us that we have a lower fraction of ubiquitinated PEX5 as the cargo addition rate increases. This reflects the higher peroxisomal PEX5 fraction, in combination with our restriction that at most one PEX5 can be ubiquitinated on each importomer. Since the peroxisomal fraction increases with the number of binding sites 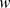, while the restriction remains unchanged, the ubiquitinated fraction decreases with increasing 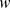.

The number of ubiquitinated PEX5 per peroxisome is shown in Fig. 3(D). The number of ubiquitin increases roughly linearly with 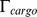 until it reaches a plateau slightly above 20 ubiquitin per peroxisome. The plateau value corresponds to the balance between ubiquitination (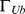) and export (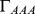). With the uncoupled and directly coupled models of translocation, neither of these processes depend on the number of PEX5 bound to an importomer — so the plateau is independent of 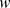. An exception is when 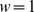, since the importomer is empty after every PEX5 export and this slightly decreases the ubiquitination rate. In comparison with the peroxisomal fraction of ubiquitinated PEX5 (Fig. 3(B)), there is a significantly larger standard deviation for the ubiquitin per peroxisome. The difference arises since each cellular fraction is averaged over 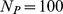 peroxisomes while ubiquitin per peroxisome is not.

### Cooperatively coupled PEX5 and ubiquitin dynamics

We have measured the same quantities for the cooperatively coupled model as for the uncoupled and directly coupled models. The cooperatively coupled results for cytosolic PEX5-cargo concentration, shown in Fig. 4(A), are very similar to those for uncoupled and directly coupled, shown in Fig. 3(A). Results with only one binding site per importomer (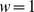) are not shown, as at least two PEX5 are needed for translocation and export with cooperative coupling.

Peroxisomal PEX5 accumulation with cooperative coupling (Fig. 4(B)) is also similar to uncoupled and directly coupled (Fig. 3(B)). One important difference is that at low cargo addition rates 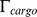 the peroxisomal PEX5 fraction vanishes for uncoupled and directly coupled but approaches a finite value (approximately 5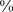) with cooperatively coupled translocation. We see from Fig. 4(B) that cooperative coupling implies a finite ratio between the peroxisomal fraction at high and low 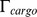, and that this ratio is controlled by the number of binding sites per importomer 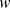. A 1∶5 ratio of PEX5∶PEX14 has been reported in normal conditions [54], and a 1∶1 ratio when PEX5 export is blocked [18]. Assuming PEX14 levels do not change with cargo traffic, these observations imply a 1∶5 ratio of PEX5 in low∶high 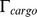 conditions, or 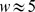 for cooperatively coupled translocation. With this choice of 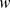, we also recover an absolute change of peroxisomal PEX5 between 5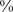 in wild-type cells to 25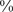 in those lacking a RING complex [53], [55]. The 1∶5 ratio is also possible with uncoupled and directly coupled models, but requires fine-tuning of 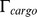.

The cooperatively coupled results for the fraction of peroxisomal PEX5 that is ubiquitinated, shown in Fig. 4(C), are also similar to those for uncoupled and directly coupled, shown in Fig. 3(C). One important difference is that the ubiquitinated peroxisomal fraction approaches 100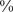 for small 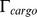 with cooperative coupling. Each importomer has at least one bound PEX5, and small 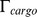 allows the bound PEX5 to be ubiquitinated long before a second PEX5 binds and allows cooperative translocation to occur.

The number of ubiquitin per peroxisome vs. the cargo addition rate 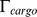, shown in Fig. 4(D) for cooperative coupling, shows strikingly different behavior from uncoupled and directly coupled translocation models. We see that the number of ubiquitin per peroxisome decreases with increasing 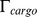. The amount of ubiquitinated PEX5 is high for low cargo addition rates because ubiquitinated PEX5 must wait for another PEX5 to arrive before it can be exported. Ubiquitinated PEX5 decreases as the cargo addition rate increases since PEX5-cargo arrives at the peroxisome more rapidly, allowing ubiquitinated PEX5 to be exported. At large 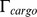, the asymptotic number of ubiquitinated PEX5 is approximately the same between the uncoupled and directly coupled, and cooperatively coupled translocation models. A slightly higher level is seen for cooperatively coupled translocation with 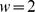, since after translocation the remaining PEX5 must wait for both ubiquitination and another PEX5 binding in the cooperative model.

Similar results have also been obtained for the five-site cooperatively coupled model without the restriction of only a single ubiquitinated PEX5 on each importomer. Fig. S1 shows that the single ubiquitin restriction does not qualitatively change the PEX5 or ubiquitin behaviours.

The cooperatively coupled model leads to high ubiquitin levels when there is little cargo addition. Since ubiquitinated peroxisomes will be degraded in mammals [13], [56] through NBR1 signalling of autophagy [12], high ubiquitin levels could be used as a degradation signal for peroxisomal disuse. We explore how a threshold level of ubiquitination could function as a trigger for specific peroxisomal autophagy (pexophagy) in greater detail below. We restrict ourselves to a five-site (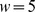) cooperatively coupled model of cargo translocation, since this recovers reported PEX5∶PEX14 stoichiometries [18], [54] and a fivefold change in peroxisomal PEX5 when RING activity is absent [55].

### Ubiquitin thresholds with cooperative coupling

A simple threshold model of pexophagy would trigger peroxisomal degradation when the number of ubiquitin on a peroxisome exceeds a certain threshold. While this appears straightforward in light of the average ubiquitin levels of Fig. 4(D), the substantial fluctuations around these averages must be considered.

To illustrate the challenge, in Fig. 5(A) we show a time-trace of the number of ubiquitin for a single peroxisome when 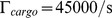 and 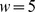 with cooperatively coupled translocation. This value of 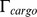 is chosen to lead to a relatively low level of ubiquitination (see Fig. 4(D)). Also shown with dashed lines are two example thresholds, at 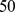 and at 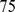 ubiquitin, which are below and above the rounded average of 58 ubiquitin. Stochastic fluctuations in the ubiquitination level lead to crossing of both thresholds.

To investigate stochastic threshold crossing more systematically, we show in Figs. 5(B) and (C) the average interval of time spent above and below various thresholds, respectively. We consider four thresholds, chosen between the minimum and maximum ubiquitin levels from Fig. 4(D), as indicated in the legend. For a given threshold, we only present data from a relatively narrow range of cargo addition rates 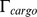. Beyond this range the threshold is only very rarely crossed, and any such crossings are very brief. This is true whether we are considering a threshold above or below the mean ubiquitin level.

The ubiquitin level is able to fluctuate over a given threshold number only for a limited range of PEX5 cargo addition rates. Within this range, the amount of time spent on either side of the threshold changes by more than three orders of magnitude. Since the range is limited, if the system is outside of the range then a simple threshold model could give a clear signal for pexophagy. Even within the range, a simple threshold model may be sufficient because the time spent on either side of the threshold changes very rapidly with changing cargo addition rate. If the pexophagy response is sufficiently slow, rapid excursions across the threshold might be ignored. It would be interesting to study how NBR1 accumulation [12] might refine this scenario.

### Varying peroxisome number with cooperative coupling

In mammals, the proliferation of peroxisomes can be stimulated by treatment with peroxisome proliferators [57]. After treatment with the proliferators is stopped the expression of peroxisomal matrix proteins (cargo) and peroxisome biogenesis factors decrease [58], [59] and the number of peroxisomes rapidly returns to normal levels [6], [7]. In mammals, 70–80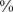 of peroxisome degradation in these circumstances is performed by autophagy [10]. Because the degradation of ubiquitinated peroxisomes is by autophagy [12], [13], [56], it is then plausible that the ubiquitin disuse signal we have proposed to signal degradation is involved in returning the peroxisome population to normal levels.

To investigate whether the ubiquitin disuse signal could be involved in returning cells to normal peroxisome levels, we have held the number of total PEX5 in our system constant and varied the number of peroxisomes, considering both a halving and doubling of the number. The peroxisomal PEX5 fraction for 50, 100, and 200 peroxisomes is shown in Fig. 6(A) and it behaves as expected: the increase from low PEX5 to high PEX5 is preserved, with the 50 peroxisome system halving and the 200 peroxisome system doubling the peroxisomal PEX5 fraction relative to the 100 peroxisome system.

As seen in Fig. 6(B), the peroxisomal ubiquitin accumulation curve is a similar shape for all three 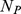, but with systematically *lower* ubiquitin accumulation for fewer peroxisomes at a given 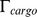. This reflects the role of PEX5-cargo traffic in clearing ubiquitin from importomers, within the cooperative coupling model of translocation. This could then provide the cell with a straightforward feedback mechanism to adjust the number of peroxisomes to match the rate of matrix protein expression. At a given 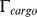 and a given ubiquitin threshold, between approximately 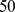 and 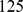 in this instance, an excess of peroxisomes would lead peroxisomes to be above the threshold and subsequently degraded. As they are degraded the ubiquitin level would decrease, until a stable number of peroxisomes was reached with ubiquitin levels below the threshold.

Given that ubiquitin signals degradation through autophagy [12], [13], [56], this mechanism is consistent with observations that autophagy is responsible for the degradation of excess peroxisomes in mammals [7]. Peroxisome proliferators increase the expression of PEX5 cargo proteins, and removing proliferators results in a decrease of cargo proteins [58], [59]. We have shown that this decrease in cargo would increase the level of ubiquitinated PEX5 on peroxisomes, and could then induce peroxisome degradation through this simple threshold model. Once decreased peroxisomal numbers reduced ubiquitin numbers below the threshold, background levels of peroxisomal biogenesis would stabilize peroxisomal numbers. Decrease of peroxisomal numbers above the threshold would occur rapidly, while increase below the threshold would be slow in the absence of a proliferation signal.

### Varying export complex number with cooperative coupling

We have been unable to determine the number of AAA export complexes on each peroxisome from the literature. Since PEX1 and PEX6 only transiently associate with peroxisomes [60] we may not have, as we assume, 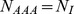. For example, the reduction in PEX26 expression during the removal of peroxisome proliferating signal [61] would result in the decrease of PEX1 and PEX6 on peroxisomes. Peroxisomal damage may also change the stoichiometry of 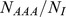.

Fig. 7(A) shows the peroxisomal PEX5 fraction vs 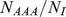 for the different 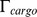 indicated by the legend. The peroxisomal PEX5 fraction is independent of larger 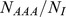 ratios, indicating that our results will not be very sensitive to our choice of 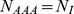. Nevertheless, at smaller ratios the peroxisomal PEX5 fraction increases as export becomes impaired. This happens first at larger 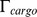, as expected.

Corresponding to PEX5 changes, the peroxisomal ubiquitin is shown in Fig. 7(B). Again, at larger 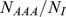 ratios the ubiquitin levels are unchanged. However, as the ratios get smaller the ubiquitin per peroxisome increases — and this happens first at higher 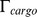. This means that if the AAA complex numbers of a particular peroxisome are significantly decreased, the ubiquitination levels of that peroxisome will increase. Nevertheless, for smaller 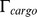 the ubiquitin levels do not change until the number of AAA complexes is below 5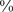 of the number of importomers. This suggests that peroxisomes may be resilient to losses of export complexes, except at high 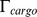.

### Summary and further discussion

We have modelled PEX5 cycling through the peroxisomal importomer, and measured the temporal dynamics of both PEX5 and ubiquitinated PEX5 associated with peroxisomes, as the matrix cargo traffic is varied via 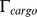. PEX5 cycling takes matrix proteins from the cytosol to the peroxisome, where they translocate into the peroxisomal matrix. However, the energetics of cargo translocation have remained unclear.

We have implemented three models of cargo translocation, illustrated in Figs. 1 and 2. The first is uncoupled cargo translocation, where the translocation of cargo happens spontaneously on PEX5-cargo association with a peroxisomal importomer. The second is directly coupled translocation, where cargo translocation happens at the same time as export of the ubiquitinated PEX5 to which the cargo is attached. The third is cooperatively coupled translocation, where cargo translocation happens at the same time as export of a different ubiquitinated PEX5 from the PEX5 to which the cargo is attached. Both directly coupled and cooperatively coupled models have cargo translocation driven by the AAA-dependent export of PEX5 from the peroxisomal membrane [28], [29].

All three translocation models have peroxisomal ubiquitin numbers that strongly depend on matrix cargo protein traffic. Both uncoupled and directly coupled translocation models have indistinguishable PEX5 and ubiquitin dynamics in which peroxisomal ubiquitinated PEX5 increases as cargo traffic increases. In contrast, cooperatively coupled translocation has decreasing levels of peroxisomal ubiquitinated PEX5 as cargo traffic increases.

Ubiquitin on the surface of peroxisomes leads to the recruitment of NBR1, which recruits the autophagic machinery [12] and leads to peroxisome degradation [12], [13]. For cooperatively coupled translocation, ubiquitin buildup at low cargo traffic could be used as a disuse signal to initiate autophagic peroxisome degradation. This feedback mechanism could be used to rapidly return peroxisome numbers to normal after induced peroxisome proliferation [7], [10], [57].

For uncoupled and directly coupled translocation models, the increase of ubiquitin levels at high cargo traffic levels means that to avoid unwanted pexophagy at high cargo traffic the autophagic response to ubiquitin must be insensitive to the maximal levels of PEX5-ubiquitin expected. This then provides a challenge to identify ubiquitinated peroxisomal membrane proteins other than PEX5 that could control pexophagy. If we assume that peroxisomal damage has a range of severity, with lightly damaged peroxisomes avoiding pexophagy, this also implies that additional pexophagy of lightly damaged peroxisomes would be quickly triggered by increases in matrix cargo traffic — as the PEX5-ubiquitin levels tipped the balance of these peroxisomes towards pexophagy.

This work investigates only the cycling and mono-ubiquitination of PEX5. We do not model the ubiquitination of other proteins or polyubiquitination of PEX5. How might these effect pexophagy signalling and/or PEX5 cycling? Polyubiquitinated PEX5 can be removed from the peroxisome membrane by the AAA complex [62], and polyubiquitinated PEX5 is targeted for degradation [19]–[21]. We assume that this background process does not significantly change PEX5 levels as cargo traffic is changed. While the ubiquitination of other peroxisomal proteins, including the polyubiquitination of PEX5, can contribute to the induction of autophagy [13], [56], we assume that these ubiquitination levels do not change significantly as cargo traffic is varied. If so, then they will simply bias or offset the PEX5 mono-ubiquitination signal and any threshold could be appropriately shifted as well. Here, we have focused on PEX5 and its accumulation on the peroxisomal membrane during changes in the import of matrix cargo. If ubiquitination of proteins other than PEX5, or polyubiquitination of PEX5, do change significantly as cargo traffic is varied, then they will need to be considered in conjunction with the PEX5 cycling of our model.

A 1∶5 ratio of PEX5∶PEX14 is observed with normal conditions [54], and a 1∶1 ratio in systems with no PEX5 export [18]. This fivefold change is also observed when peroxisomal PEX5 goes from 5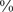 in wild-type to 25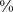 in cells without a functional RING complex [53], [55], implying no ubiquitination and so no export. It is possible to recover this fivefold change with uncoupled and directly coupled translocation, but only by tuning parameters – and only for specific 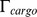 values. These ratios are more naturally recovered for a five-site importomer with cooperatively coupled translocation because with cooperative coupling the importomer cannot remove all PEX5. The 1∶5 ratio would then correspond to low cargo traffic, and the 1∶1 ratio to high cargo traffic or no export.

Miyata *et al* [63] were able to measure peroxisome associated PEX5 and ubiquitinated-PEX5. Our modelling indicates that PEX5 cycling responds in just a few seconds to changes in matrix cargo traffic. This response is much faster than timescales to change other protein expression or peroxisome numbers, so we expect that changes in peroxisomal ubiquitin with traffic could directly distinguish between the contrasting predictions of uncoupled or directly coupled translocation models and cooperatively coupled translocation models. From Fig. 3(D) and Fig. 4(D), we see that in the linear regime a doubling of matrix cargo traffic leads to a doubling of peroxisomal PEX5-ubiquitin for uncoupled or directly coupled models, and a halving of peroxisomal PEX5-ubiquitin for the cooperatively coupled model. Complicating this is that we might expect to be close to the end of the linear regime (i.e. 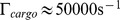) in normal conditions, so that the linear response would be seen only for a marked decrease of matrix cargo traffic. Nevertheless, we might expect to be in the linear regime after induced peroxisomal proliferation and before pexophagy has reduced the number of peroxisomes significantly.

Our model is tuned for mammalian peroxisomes, since the E2 enzyme for monoubiquitination of PEX5 is cytosolic and is embodied in our model via a 3d diffusion-limited rate 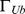 from Eqn. 1. In yeast, the E2 for monoubiquitination of Pex5 is Pex4, which is attached to the peroxisome membrane by Pex22 so that 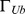 should be determined by a 2d diffusion-limited rate from Eqn. 2. We do not expect any qualitative changes to the Pex5 cycling because of this, and cooperatively coupled translocation should lead to an increase of ubiquitinated Pex5 in yeast when matrix cargo traffic is reduced. This could be used to probe the translocation mechanism of peroxisomal matrix proteins in yeast. Nevertheless, the role of peroxisomal ubiquitin in pexophagy appears to be, at best, indirect in yeast [10], [64]–[66] so that our discussion of ubiquitin thresholds and pexophagy is restricted to mammalian systems.

## Supporting Information

Figure S1**Allowing multiple ubiquitin per importomer, with cooperative coupling.** We generally impose a restriction that each importomer have at most one ubiquitinated PEX5. Here we relax this restriction for the cooperatively coupled 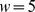 site model, and allow all bound PEX5 to be ubiquitinated. Blue squares are the same data as Fig. 3, with at most one ubiquitinated PEX5. Orange triangles are without the restriction, and show qualitatively similar behavior. (A) Cytosolic PEX5-cargo concentration vs. PEX5 cargo addition rate, 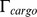. The dashed black line is the measured cytosolic PEX5 concentration of 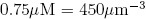 [43]. (B) peroxisomal PEX5 fraction vs. 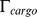. (C) Fraction of peroxisomal PEX5 that is ubiquitinated vs. 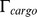. (D) ubiquitin per peroxisome vs. 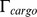.(TIFF)

Figure S2**Distribution of time intervals below ubiquitination threshold.** Frequency distribution of time intervals spent below a threshold of 100 ubiquitin for the cooperatively coupled five-site model with 100 peroxisomes and 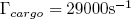. Data is taken for one simulated minute. A characteristic bimodal distribution is seen.(TIFF)
