# Supplementary figures and images for: PEX5 and Ubiquitin Dynamics on Mammalian Peroxisome Membranes
Source: PLoS Comput Biol. 2014 Jan 16;10(1):e1003426. doi: 10.1371/journal.pcbi.1003426 (PMC3894153; doi:10.1371/journal.pcbi.1003426)

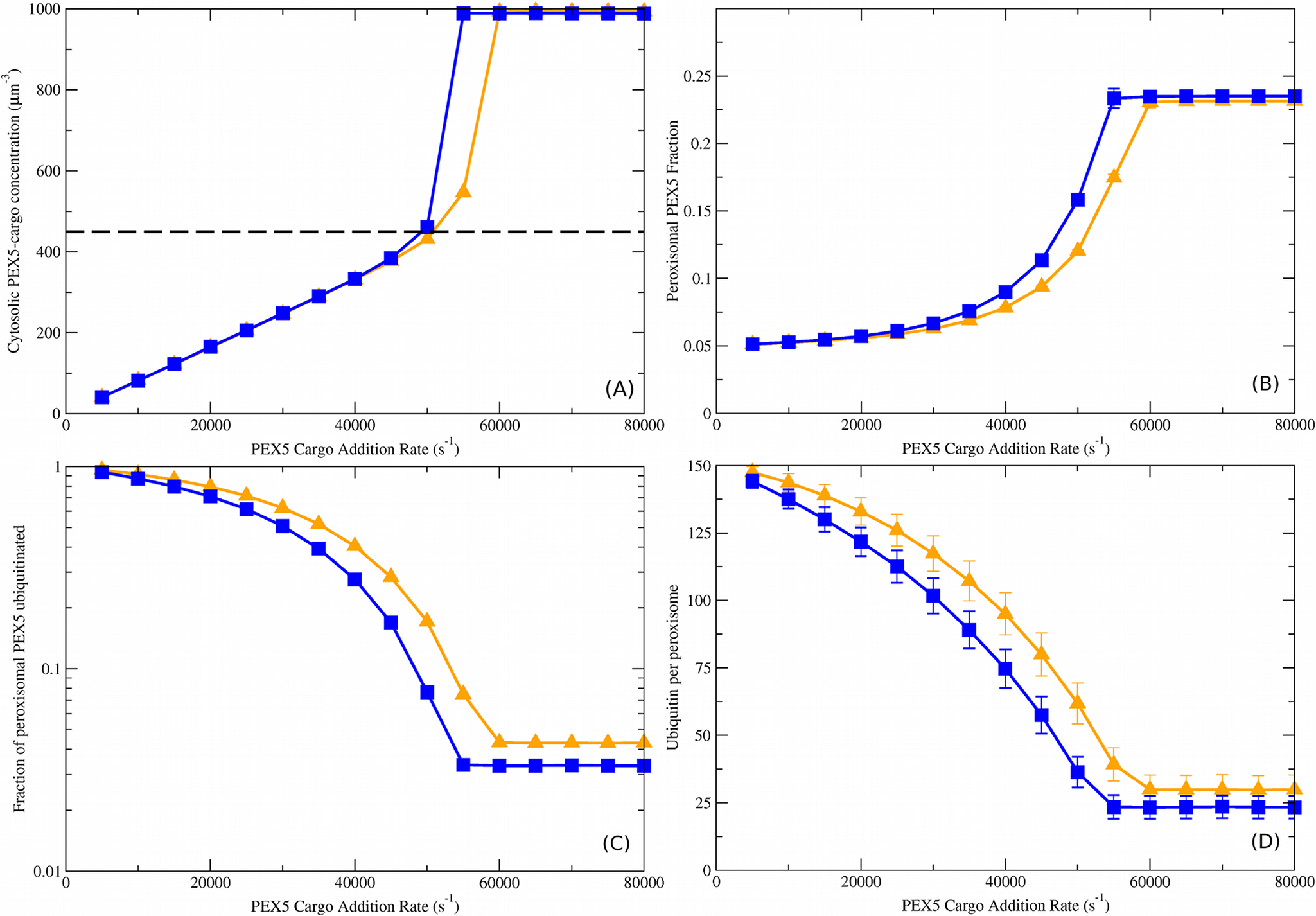

Supplement: Figure S1 — Allowing multiple ubiquitin per importomer, with cooperative coupling. We generally impose a restriction that each importomer have at most one ubiquitinated PEX5. Here we relax this restriction for the cooperatively coupled site model, and allow all bound PEX5 to be ubiquitinated. Blue squares are the same data as Fig. 3, with at most one ubiquitinated PEX5. Orange triangles are without the restriction, and show qualitatively similar behavior. (A) Cytosolic PEX5-cargo concentration vs. PEX5 cargo addition rate, . The dashed black line is the measured cytosolic PEX5 concentration of [43]. (B) peroxisomal PEX5 fraction vs. . (C) Fraction of peroxisomal PEX5 that is ubiquitinated vs. . (D) ubiquitin per peroxisome vs. . (TIFF) [file pcbi.1003426.s001.tiff]

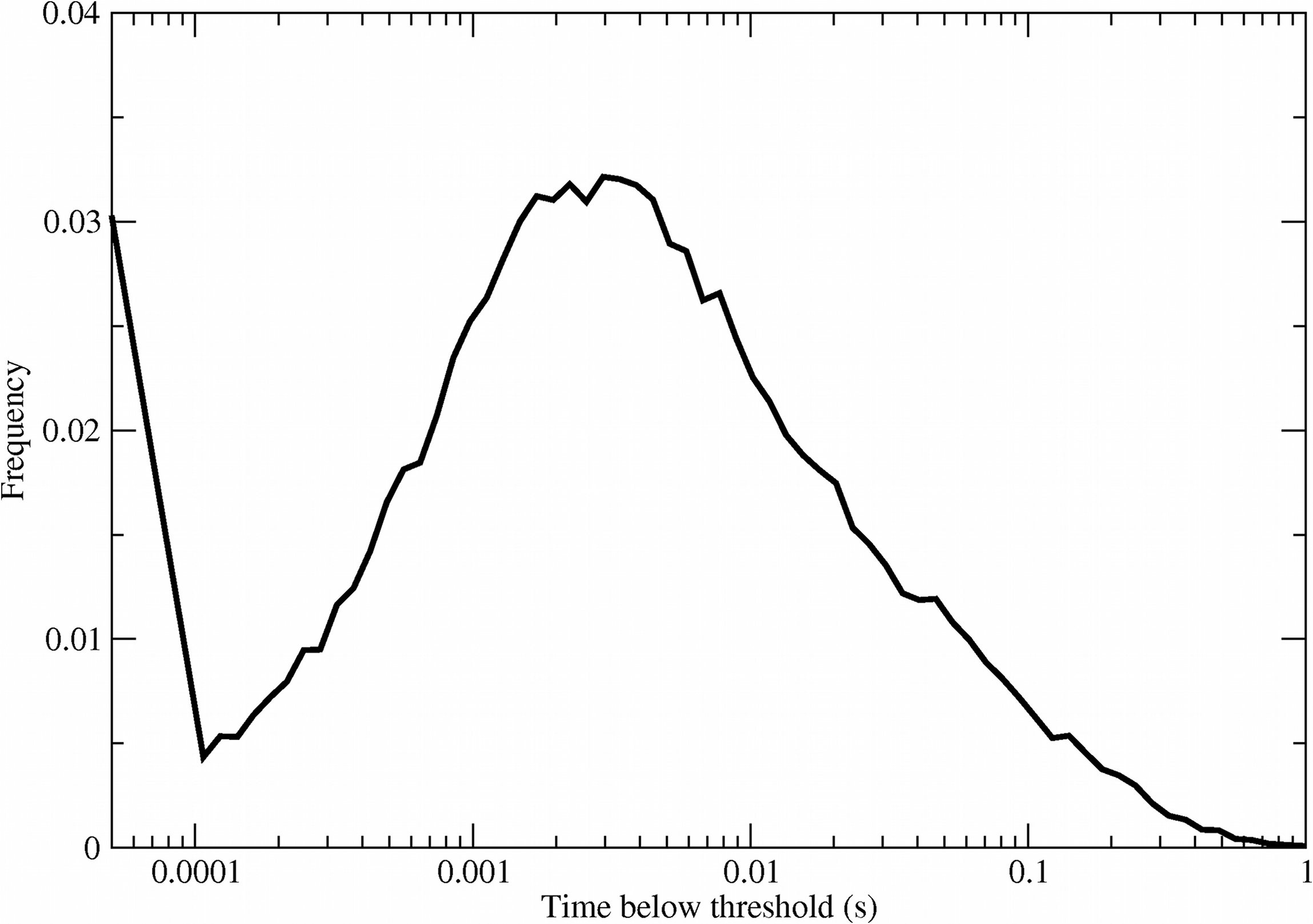

Supplement: Figure S2 — Distribution of time intervals below ubiquitination threshold. Frequency distribution of time intervals spent below a threshold of 100 ubiquitin for the cooperatively coupled five-site model with 100 peroxisomes and . Data is taken for one simulated minute. A characteristic bimodal distribution is seen. (TIFF) [file pcbi.1003426.s002.tiff]
